# Small Vessel Disease Burden and Outcomes of Mechanical Thrombectomy in Ischemic Stroke: A Systematic Review and Meta-Analysis

**DOI:** 10.3389/fneur.2021.602037

**Published:** 2021-04-07

**Authors:** Tao Xu, You Wang, Jinxian Yuan, Yangmei Chen, Haiyan Luo

**Affiliations:** Department of Neurology, The Second Affiliated Hospital of Chongqing Medical University, Chongqing, China

**Keywords:** small vessel disease, thrombectomy, ischemic stroke, outcome, meta-analysis

## Abstract

**Background:** Cerebral small vessel disease (SVD) is prevalent in the population, especially among elderly individuals. Substantial uncertainties remain about the clinical relevance of SVD with outcomes of mechanical thrombectomy (MT) in acute ischemic stroke (AIS).

**Objectives:** This systematic review and meta-analysis was performed to evaluate the association between SVD and clinical outcomes in patients with AIS undergoing MT.

**Methods:** We systematically searched the Medline, Embase, and Cochrane databases for relevant clinical studies. The exposure of SVD mainly included leukoaraiosis, cerebral microbleeds (CMBs), and lacunes. The pooled OR was used to calculate the association between each subtype of SVD and outcomes of MT. The primary outcome was poor functional outcome, which was defined as a modified Rankin Scale score (mRS) ≥3 at 90 days after MT. The secondary outcomes included mortality at 90 days, in-hospital mortality, intracranial hemorrhage (ICH) and symptomatic intracranial hemorrhage (sICH), successful recanalization and futile recanalization (FR), early neurological improvement, and early neurological deterioration (END) after MT.

**Results:** Overall, 20 studies with 5,189 patients with AIS undergoing MT were included. High leukoaraiosis burden (HLB) at baseline was associated with increased risks of poor functional outcome at 90 days (OR 2.70, 95% CI 2.01–3.63; *p* < 0.001; 10 studies; *n* = 2,004), in-hospital mortality (OR 4.06, 95% CI 1.48–11.13; *p* = 0.006; 2 studies; *n* = 314), FR (OR 5.00, 95% CI 2.86–8.73; *p* < 0.001; 3 studies; *n* = 493), and END (OR 2.65, 95% CI 1.09–6.45; 1 study; *n* = 273) after MT. HLB (VSS 3–4 or FS ≥ 2) at baseline was not associated with mortality at 90 days, ICH, or sICH after MT. CMBs at baseline were found to be associated with increased risks of poor functional outcome at 90 days (OR 1.84, 95% CI 1.17–2.90; *p* = 0.008; 2 studies; *n* = 1,924) after MT. We found no association between the presence of lacunes and poor functional outcome at 90 days after MT.

**Conclusions:** In patients with AIS undergoing MT, HLB and CMBs were associated with increased risks of unfavorable outcomes after MT.

## Introduction

Cerebral small vessel disease (SVD) is prevalent in the population, especially among the elderly (1). SVD is a disorder of cerebral small vessels, causing various lesions that can be detected with CT or MRI scans (2). The pathogenesis of SVD is complex, and it is thought to result from abnormalities in cerebral arterioles, capillaries, and venules (2). Typical SVD lesions mainly include leukoaraiosis, presumably due to vascular origin, cerebral microbleeds (CMBs), lacunes, perivascular spaces, and lacunar infarcts (1, 2). Most SVD is silent and sporadic, which can be aggravated by increased SVD lesions, further resulting in secondary degeneration in the cerebral cortex or brainstem and ultimately giving rise to whole brain effects (2). Thus, SVD is considered a dynamic, global brain disorder that has been associated with increased risks of stroke, cognitive decline, dementia, movement disorder, and even death (1, 2). SVD is more prevalent in patients with ischemic stroke than in the general population (3). In acute ischemic stroke (AIS), patients with SVD undergoing intravenous thrombolysis (IVT) therapy may have less favorable functional outcomes and experience more frequent intracranial hemorrhage (ICH) events (4). SVD, therefore, is considered a marker of poor prognosis in IVT for AIS.

The efficacy and safety of mechanical thrombectomy (MT) in the treatment of AIS caused by cerebral large vessel occlusion (LVO) have been confirmed by clinical studies over the past several years (5). MT is therefore considered a standard therapy for AIS caused by LVO in clinical practice. To date, although a number of clinical studies have investigated the association between SVD and outcomes of MT in AIS, the results have been inconsistent, possibly due to the small sample sizes and the varying definitions of SVD and AIS outcomes in these studies. Hence, substantial uncertainties remain about the magnitude of the association between SVD and outcomes of MT in AIS, which is worthy of further exploration. The aim of this systematic review and meta-analysis was to evaluate the association between SVD and outcomes in patients with AIS undergoing MT.

## Materials and Methods

### Search Strategy

This meta-analysis was performed according to the meta-analysis of observational studies in epidemiology (MOOSE) guidelines (6). We systematically searched the literature in the Medline, Embase, and Cochrane Central Register of Controlled Trials (CENTRAL) databases using a predefined search strategy (the detailed search terms and strategy are available in Supplementary Table 1). We also examined the reference lists of the included articles to obtain additional relevant studies. There was no limitation on literature language or publication type or time. The last search was conducted in June 2020. Institutional ethics committee approval did not apply to this study.

### Inclusion Criteria

Studies were included if they met all of the following inclusion criteria: (1) Population: The study population included patients with AIS undergoing MT; (2) Exposure and outcome: The study explored the associations between SVD at baseline and the outcomes of MT in AIS; (3) SVD and MT assessments: SVD was defined as leukoaraiosis or white matter hyperintensity (WMH), lacunes, and CMBs assessed with neuroimaging techniques, including CT or MRI scan; MT was defined as endovascular thrombectomy using a stent retriever or an aspiration technique for the treatment of AIS; (4) Outcome assessment: The study reported the adjusted or unadjusted ORs and the corresponding 95% CIs for the magnitude of the association between SVD subtypes and the outcomes of MT or provided raw data that could be used to calculate ORs and 95% CIs; (5) Design: The study had a cohort, case–control, or controlled trial design; (6) Article availability: The study included original research with full-article availability.

We excluded non-original articles, articles with insufficient data or irrelevant outcomes, and case reports from the selected studies.

### Study Screening and Selection

A literature search was conducted independently by two authors (TX and YW) according to the selection criteria. First, two reviewers identified studies for full-article review based on titles and abstracts. Then, full-text articles were reviewed if one or both reviewers considered a study potentially eligible. The reference lists of retrieved articles were manually screened to identify additional relevant studies. We resolved disagreements about the inclusion of a study by discussion until a consensus was reached. If there was a potentially significant sample overlap among multiple studies, the study with the longest follow-up or highest sample size was included.

### Data Extraction and Qualitative Assessment

A predefined spreadsheet was used to extract the following data from each article: first author, publication year, study period and design, methods of MT (stent retriever or aspiration technique), vascular lesion sites, population demographics, subtypes of SVD and their assessment strategies, and outcomes of MT. Then, the ORs and 95% CIs or raw data were extracted to calculate ORs for the association between SVD and outcomes of MT. The OR was extracted from the most fully adjusted model when a study reported both unadjusted and adjusted ORs. When the effect estimate was not directly provided, ORs and 95% CIs were calculated based on available data.

The Newcastle–Ottawa scale was used to assess the quality of the included studies (7). The full score was 9 stars, and a high-quality study was defined as a study awarded ≥8 stars.

### Exposure Assessments and Outcome Definitions

The SVD assessment strategies of the included studies are summarized in Table 1. Leukoaraiosis and WMH were assessed with the van Swieten scale (VSS) (28), the Fazekas scale (FS) (29), or volumetry with MRICro software (16). Leukoaraiosis and WMH, as reported by the studies included in this meta-analysis, have a similar definition and assessment strategy. Therefore, WMHs were considered equally to leukoaraiosis when pooled estimates were performed. The exposure of leukoaraiosis was dichotomized as high burden or low burden. High leukoaraiosis burden (HLB) was defined as moderate to severe leukoaraiosis with semi-quantitative severity scales (VSS 3–4 or FS ≥2) or greater volume of WMH assessed with MRICro software. CMBs were assessed with the Microbleed Anatomical Rating Scale (MARS) (30). CMB was defined as a round or ovoid small area (<10 mm in diameter) of signal loss on T2-weighted gradient-echo imaging or susceptibility-weighted imaging. CMB burden was defined as the presence of cerebral CMBs (≥1) detected with an MRI scan. Lacunes were defined as round or ovoidal hypodense lesions ≤20 mm in diameter in the basal ganglia, white matter, or brainstem detected by CT scan at the time of AIS (17). Lacune burden was defined as the presence of multiple lacunes (≥2) in the brain detected with a CT or MRI scan. The definitions of HLB, CMB burden, and lacune burden of the included studies are listed in Supplementary Table 2.

**Table 1:** Characteristics of the studies included in the meta-analysis.

**References**	**Country**	**Study period**	**Study design**	**Primary methods of MT**	**Vascular lesion sites**	**No. in cohort/age, years (median with range or mean with SD)/men, %**	**Subtypes of SVDs**	**Assessment of SVDs**	**Outcome**
Benson et al. (8)	USA	NA	R	NA	NA	174/68.0 (9.1)/48.9%	Leukoaraiosis	FS on CT scan	90-day mRS, FR, and ICH
Mutzenbach et al. (9)	Austria	2012–2019	R	SR and AT	ACC	209/75 (63–81)/46.9%	Leukoaraiosis	Self-report on CT or MRI scan	90-day mRS, in-hospital death, sICH, and ICH
Mistry et al. (10)	USA	2017–2018	P	NA	ACC	389/68.0 (58.0–79.0)/50.0%	WMH	VSS on CT scan	90-day mRS, 90-day death, sICH, ENI, and SRE
Mikati et al. (11)	USA	2012–2016	R	SR	ACC	144/68.0 (57.0–81.0)/51.0%	Leukoaraiosis	VSS on CT scan	90-day mRS
Mechtouff et al. (12)	France	2013–2019	R	NA	ACC	293/67.1 (16.2)/54.6%	WMH	FS on FLAIR MRI imaging	90-day mRS, ICH, and SRE
Guo et al. (13)	China	2014–2017	R	SR	ACC	273/64.4 (11.9)/63.0%	Leukoaraiosis	VSS on CT or MRI imaging	ENI and END
Liu et al. (14)	China	2015–2017	R	SR	ACC	97/70.0 (12.4)/61.9%	Leukoaraiosis	FS on FLAIR MRI imaging	90-day mRS, 90-day death, and sICH
Guo et al. (15)	China	2014–2017	R	SR	ACC	251/64.4 (11.8)/62.2%	Leukoaraiosis	VSS on CT or MRI imaging	90-day mRS, 90-day death, FR, and sICH
Boulouis et al. (16)	France	2015–2018	R	SR and AT	ACC	496/68.1 (15.0)/50.0%	WMH	Volumetry with MRICro software	90-day mRS, 90-day death, and sICH
Arba et al. (17)	Italy	2015–2017	R	NA	ACC	175/72.3 (12.4)/51.0%	Leukoaraiosis and Lacunes	VSS and self report on CT scan	90-day mRS
Sillanpaa et al. (18)	Finland	2013–2014	P	SR	ACC	68/70.6 (8.8)/53.0%	Lacunes	Self report on CT scan	90-day mRS
Choi et al. (19)	South Korea	2007–2017	P	SR	NA	1532/NA/55.8%	CMBs	MARS on T2-GRE MRI	90-day mRS and sICH
Gilberti et al. (20)	Italy	2012–2016	R	SR and AT	ACC	68/74.0 (66.0–79.0)/50.0%	Leukoaraiosis	VSS on CT or MRI imaging	FR
Atchaneeyasakul et al. (21)	USA	2012–2015	R	SR	ACC	56/67.3 (14.2)/53.6%	WMH	Volumetry with MRICro software	90-day mRS, 90-day death, ICH, and SRE
Shi et al. (22)	China	2002–2012	P	SR and AT	ACC and PCC	206/66.8 (17.6)/42.2%	CMBs	MARS on T2-GRE MRI	In-hospital death and ICH
Giurgiutiu et al. (23)	USA	2007–2009	R	SR and AT	ACC	73/67.2 (15.7)/43.8%	Leukoaraiosis	Volumetry with MRICro software	90-day mRS
Zhang et al. (24)	USA	2006–2013	R	NA	NA	129/71.0 (58.0–80.0)/60%	Leukoaraiosis	VSS on CT scan	90-day mRS
Gratz et al. (25)	Switzerland	2010–2013	R	NA	ACC and PCC	392/68.1 (13.7)/56.9%	CMBs	An assessment tool on MRI-SWI	90-day mRS, 90-day death, sICH, and ICH
Soize et al. (26)	France	2010–2012	R	SR	ACC	59/63.0 (16.0)/45.8%	Leukoaraiosis	FS on FLAIR MRI imaging	90-day mRS, 90-day death, and sICH
Shi et al. (27)	China	2002–2008	R	SR	ACC	105/65.9 (18.9)/NA	Leukoaraiosis	FS on FLAIR MRI imaging	In-hospital death and ICH

*MT, mechanical thrombectomy; P, prospective; R, retrospective; SR, stent retriever; AT, aspiration technique; ACC, anterior cerebral circulation; PCC, posterior cerebral circulation; SVD, small vessel disease; WMH, white matter hyperintensity; CMBs, cerebral microbleeds; NA, not available; VSS, van Swieten scale; FS, Fazekas scale; mRS, modified Rankin Scale scores; MARS, the Microbleed Anatomical Rating Scale; FLAIR, fluid-attenuated inversion recovery; GRE, gradient-recall echo; SWI, susceptibility-weighted imaging; sICH, symptomatic intracranial hemorrhage; ICH, intracranial hemorrhage; SRE, successful recanalization; FR, futile recanalization; ENI, early neurological improvement; END, early neurological deterioration*.

The primary outcome was poor functional outcome, which was defined as a modified Rankin Scale score (mRS) ≥3 at 90 days after MT. The secondary outcomes included mortality at 90 days after MT, in-hospital mortality, ICH and symptomatic ICH (sICH) after MT, successful recanalization and futile recanalization (FR) with MT, and early neurological improvement (ENI) and early neurological deterioration (END) after MT. For secondary outcomes, ICH after MT was defined as any hemorrhage, including hemorrhagic infarction, parenchymal hemorrhage within or outside infarcted tissue, or intracranial–extracerebral hemorrhage, which was detected with CT or MRI scan after MT (31). sICH was defined as post-MT ICH with significant neurological deterioration (National Institutes of Health Stroke Scale (NIHSS) scores ≥ 4 in total) (31). Successful recanalization was defined as a modified Thrombolysis in Cerebral Infarction (mTICI) score of 2b−3 after MT (32). FR was defined as successful recanalization with poor functional outcome at 90 days after MT (20). ENI was defined as a decrease of ≥4 points on the NIHSS or a NIHSS score of 0–1 within 24 h after baseline assessment (13). END was defined as an increase of ≥4 points on the NIHSS within 24 h after baseline assessment (13).

### Statistical Analysis

The pooled ORs were used to evaluate the associations between SVD and outcomes of MT. To adjust multiple comparisons for evaluating the association between SVD and each outcome of MT, a *p*-value less than the Bonferroni-corrected *p*-value threshold was considered statistically significant. The magnitude of heterogeneity between estimates was quantified with the *I*^2^ heterogeneity test statistic in this meta-analysis (33). Then, to find the sources of heterogeneity between estimates, potential diversities were checked (e.g., adjustments for key confounders, assessment strategies of SVD, and varied recanalization rates among the included studies), and subgroup analyses restricted to these predefined variables were performed. The pooled estimates and 95% CIs were calculated with a random-effects model due to significant between-study heterogeneity from heterogeneity analyses. Moreover, meta-regression analyses were performed to investigate the influence of the predefined variables on the heterogeneity of the studies, and P_interaction_ was used to assess the heterogeneity between subgroups. Publication bias was investigated visually with funnel plots and statistically with Egger's tests (34) when a pooled estimate included ≥5 studies. We used “trim and fill” analysis to adjust the pooled effect estimate for detected publication bias (35). In heterogeneity, meta-regression, and publication bias analyses, *p*-value < 0.050 was considered statistically significant. STATA version 12.0 (StataCorp, College Station, TX, USA) was used for the statistical analyses.

## Results

### Study Characteristics and Qualitative Assessment

**Figure 1:**
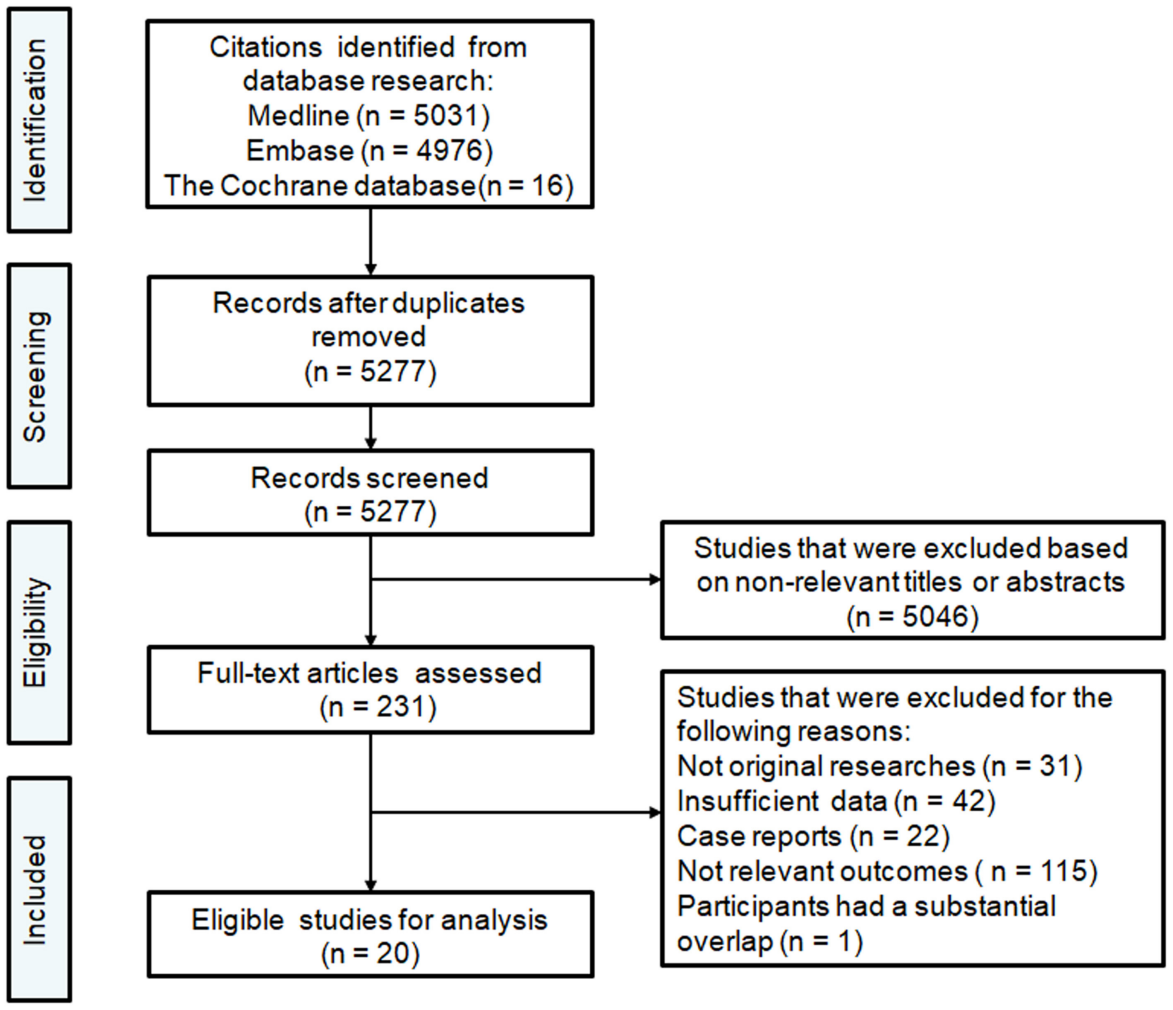
Flowchart of the literature search process.

A flow chart of the selection procedure is illustrated in Figure 1. The initial literature search provided 5,277 unduplicated records that were screened for eligibility. Of the 5,277 records assessed with titles and abstracts, the full texts of 231 articles were reviewed. Overall, 20 articles published between 2012 and 2020 met our inclusion criteria and were finally included in this meta-analysis (8–27). The study characteristics are summarized in Table 1. Of 20 studies with 5,189 patients, 16 had a retrospective design, and four had a prospective design. All studies reported that they performed endovascular thrombectomy for the treatment of AIS, and 14 studies reported methods of MT, including stent retrievers (Solitaire, Trevo, and Merci were the most often-used devices) and aspiration techniques (the Penumbra aspiration system was the most often-used device). Seventeen studies reported vascular lesion information: 15 included patients with AIS due to LVO in the anterior cerebral circulation involving the proximal middle cerebral artery, the internal carotid artery, and the terminal carotid artery, and two included patients with AIS due to LVO in the anterior or posterior cerebral circulation involving the basilar artery and the vertebral artery. Except for the study conducted by Choi and colleagues (comprising 1,532 patients) (19), the included studies had relatively small sample sizes, ranging from 56 to 496 patients. In all included studies, the majority of the included patients were elderly. Of the 20 included studies, 16 explored the clinical relevance between leukoaraiosis and the outcomes of MT (8–17, 20, 21, 23, 24, 26, 27). Three studies investigated the association between CMBs and outcomes of MT (19, 22, 25), and two studies explored the association between lacunes and outcomes of MT (17, 18).

For the quality assessment of the included studies (summarized in Supplementary Table 3), the mean score of the included studies was 7.00 (range: 5–9), and seven were considered to have high study quality.

### Leukoaraiosis and Outcomes of MT

**Figure 2:**
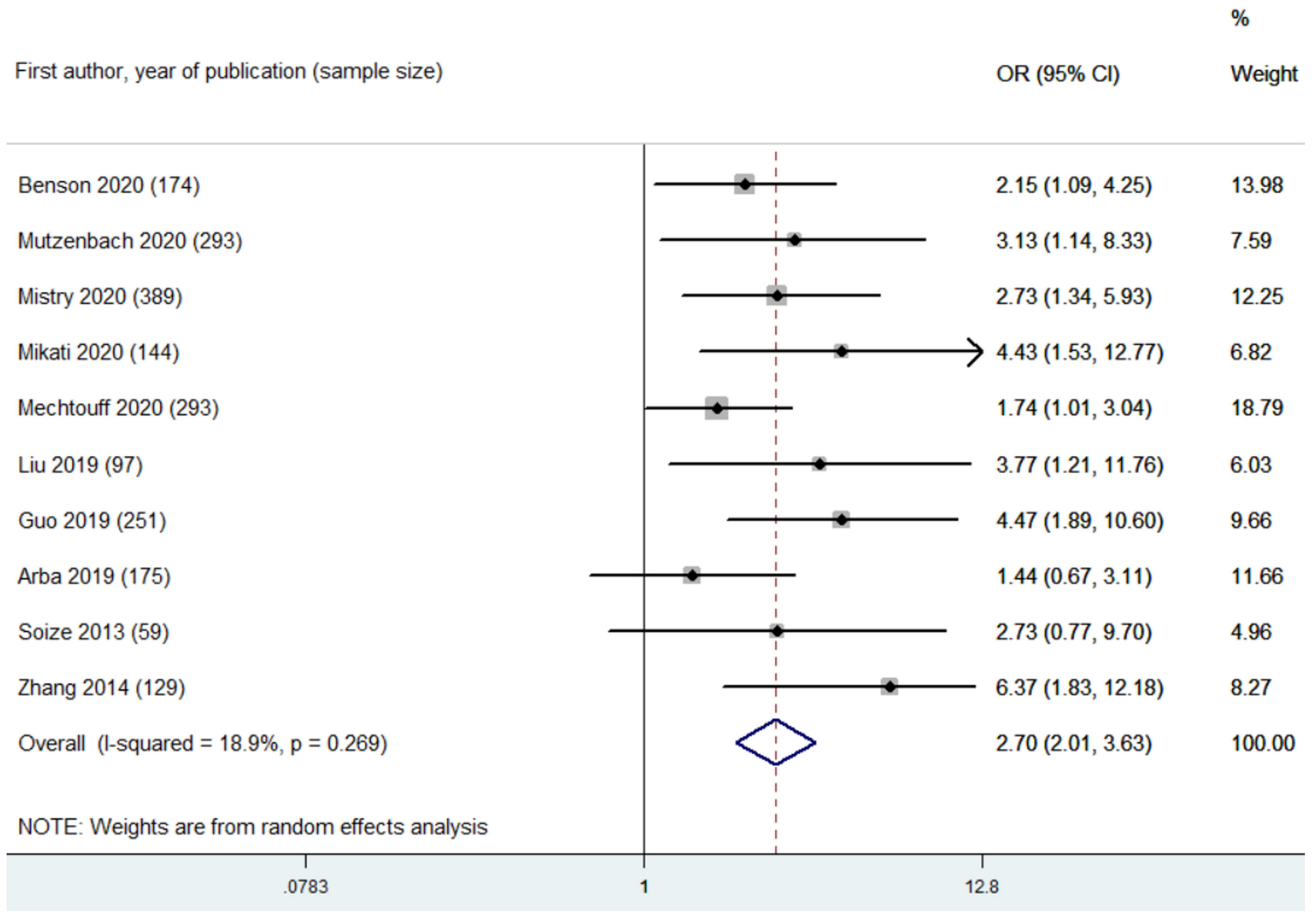
Summary of ORs for the association between high leukoaraiosis burden and poor functional outcome at 90 days.

**Figure 3:**
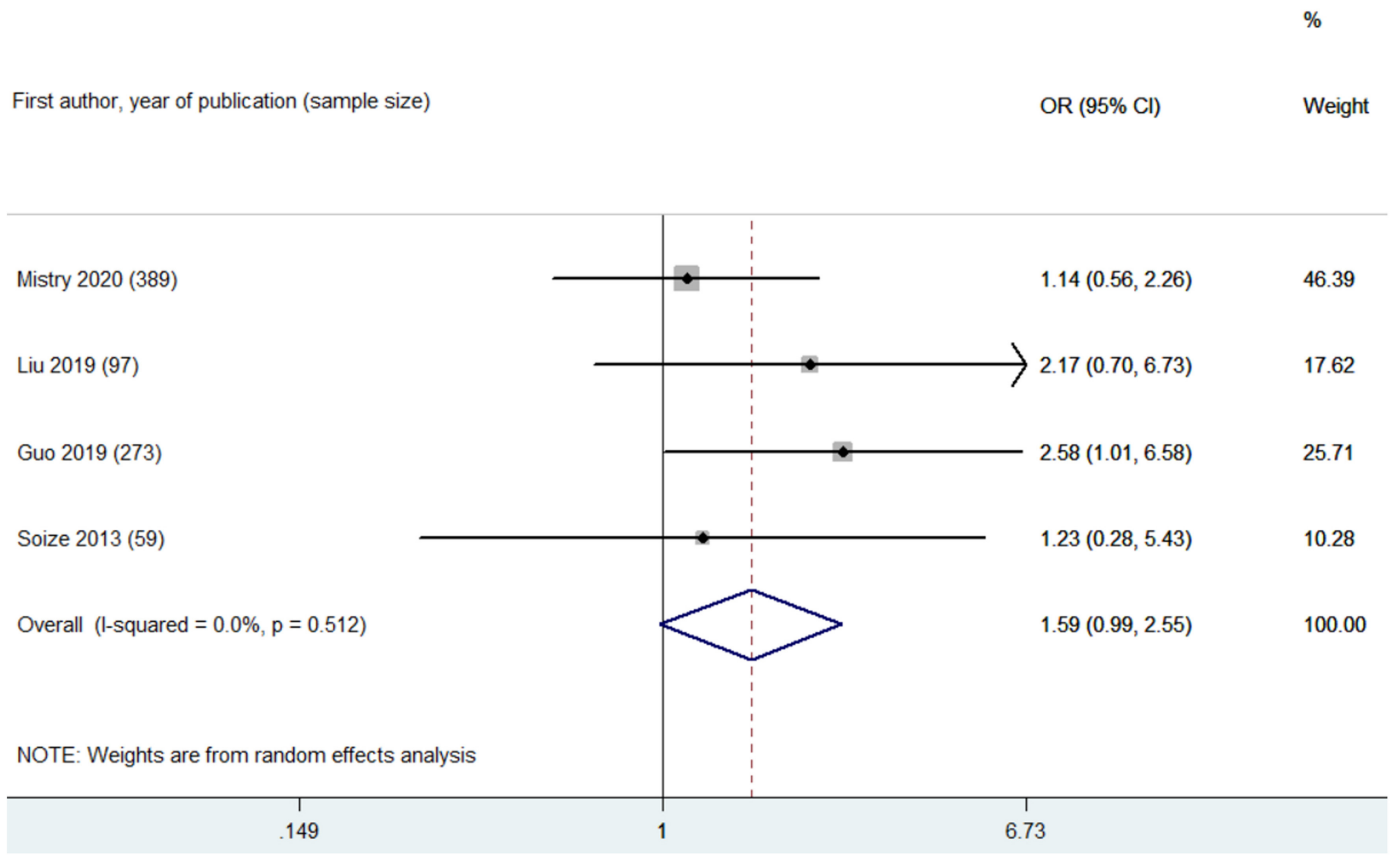
Summary of ORs for the association between high leukoaraiosis burden and mortality at 90 days.

**Figure 4:**
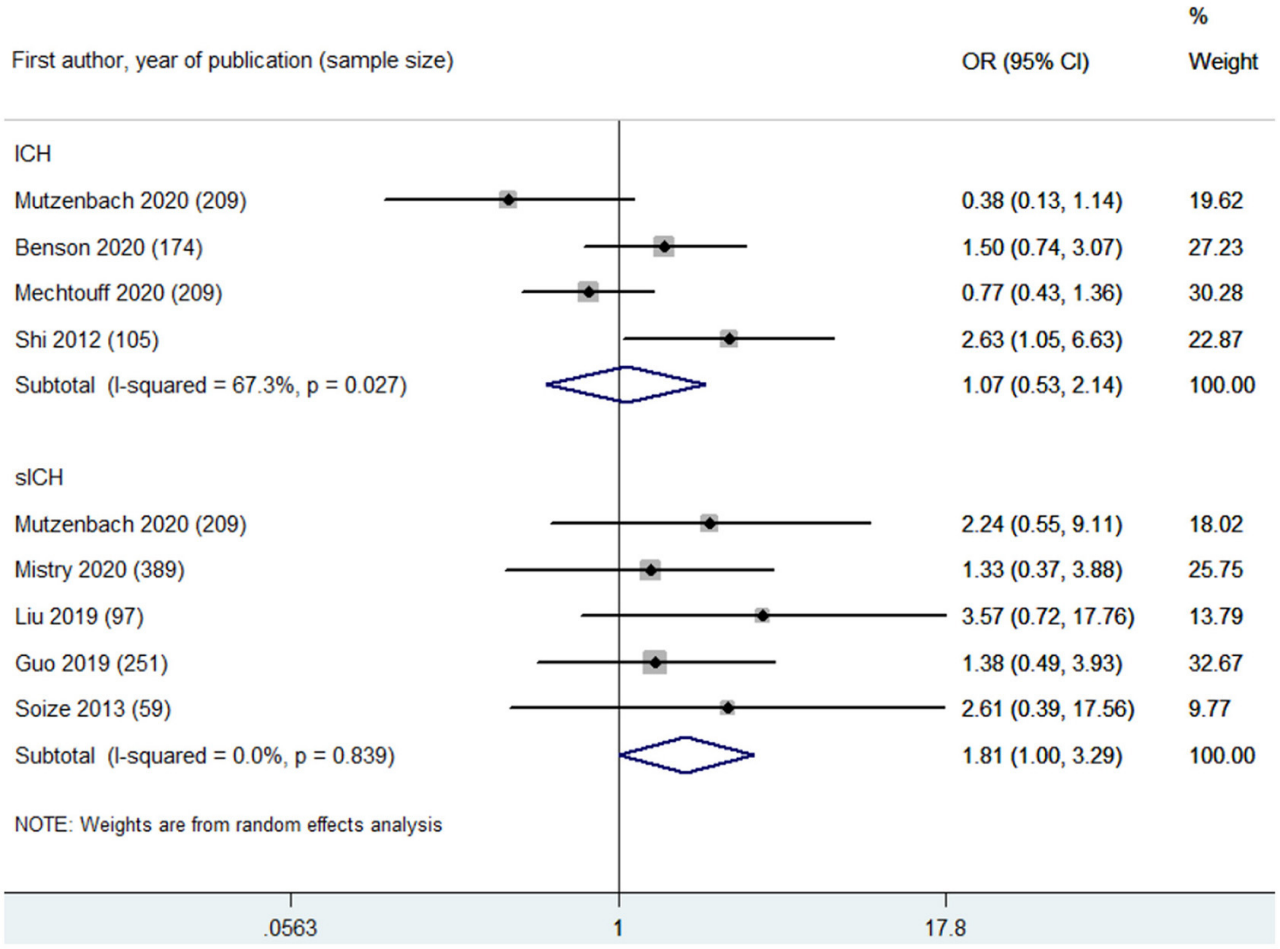
Summary of ORs for the associations between high leukoaraiosis burden and intracranial hemorrhage (ICH) and symptomatic intracranial hemorrhage (sICH) after MT.

Based on leukoaraiosis severity as assessed with semi-quantitative scales (VSS 3–4 or FS ≥2), HLB was associated with an increased risk of poor functional outcome at 90 days (OR 2.70, 95% CI 2.01–3.63; *p* < 0.001; 10 studies) (Figure 2). For secondary outcomes, HLB was not associated with mortality at 90 days (OR 1.59, 95% CI 0.99–2.55; *p* = 0.056; 4 studies) (Figure 3); however, HLB was associated with an increased risk of in-hospital mortality (OR 4.06, 95% CI 1.48–11.13; *p* = 0.006; 2 studies) (Supplementary Table 4). For post-MT ICH, HLB was not associated with post-MT ICH (OR 1.07, 95% CI 0.53–2.14; p = 0.858; 4 studies) or post-MT sICH (OR 1.81, 95% CI 1.00–3.29; *p* = 0.051; 5 studies) (Figure 4). For recanalization grade with MT, HLB was not associated with successful recanalization (OR 1.16, 95% CI 0.73–1.86; *p* = 0.528; two studies) (Supplementary Table 4). However, patients with HLB had a higher risk of experiencing FR (OR 5.00, 95% CI 2.86–8.73; *p* < 0.001; three studies) (Supplementary Table 4). For early outcomes after MT (Supplementary Table 4), HLB was not associated with ENI (OR 0.58, 95% CI 0.19–1.72; *p* = 0.324; 2 studies). It was, however, associated with an increased risk of END after MT (OR 2.65, 95% CI 1.09–6.45; one study).

Based on leukoaraiosis severity as assessed with volumetric software, HLB was associated with an increased risk of poor functional outcome at 90 days (OR 1.04, 95% CI 1.02–1.07; *p* = 0.001; 3 studies) (Supplementary Table 4). For secondary outcomes (Supplementary Table 4), HLB was not associated with mortality at 90 days (OR 1.13, 95% CI 0.79–1.62; *p* = 0.515; 2 studies). For post-MT ICH, HLB was not associated with post-MT ICH (OR 1.06, 95% CI 0.60–1.87; one study) or post-MT sICH (OR 0.99, 95% CI 0.93–1.04; 1 study). For recanalization grade with MT, HLB was not associated with successful recanalization (OR 1.30, 95% CI 0.70–2.38; one study).

### CMBs and Outcomes of MT

**Figure 5:**
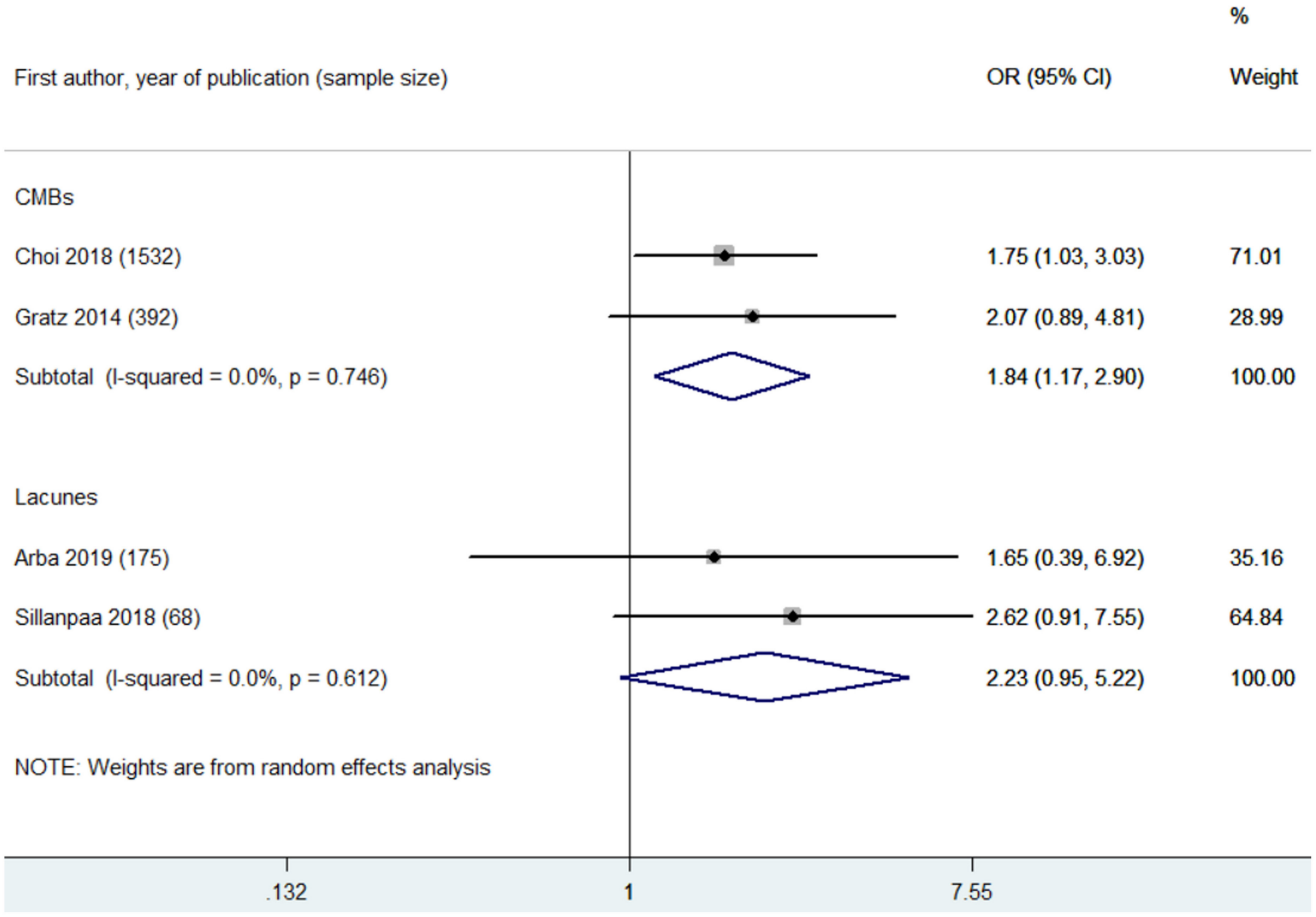
Summary of ORs for the association between cerebral microbleeds (CMBs) and poor functional outcome at 90 days, and summary of ORs for the association between lacunes and poor functional outcome at 90 days.

CMBs were found to be associated with increased risks of poor functional outcome (OR 1.84, 95% CI 1.17–2.90; *p* = 0.008; two studies) (Figure 5) and mortality (OR 2.28, 95% CI 1.04–4.99; 1 study) at 90 days (Supplementary Table 4). However, CMB burden was not associated with in-hospital mortality (OR 0.57, 95% CI 0.17–1.84; 1 study) (Supplementary Table 4). CMB burden was not associated with post-MT ICH (OR 0.60, 95% CI 0.27–1.32; *p* = 0.204; two studies) or post-MT sICH (OR 1.42, 95% CI 0.19–10.55; *p* = 0.733; 2 studies) (Supplementary Table 4).

### Lacunes and Outcomes of MT

Two eligible studies reported the association between lacunes and poor functional outcome at 90 days after MT (17, 18). Lacune burden was not associated with poor functional outcome at 90 days (OR 2.23, 95% CI 0.95–5.22; *p* = 0.066; 2 studies) (Figure 5).

### Heterogeneity and Sensitivity Analyses

For primary outcome, low heterogeneity was found in the pooled estimate of the association between HLB (assessed with VSS 3–4 or FS ≥ 2) and poor functional outcome at 90 days (*I*^2^ = 18.90%, p of heterogeneity = 0.269) (Figure 2). Omitting each study in turn did not significantly change the results or heterogeneity. Adjustments for confounders in each included study were considered as key variables affecting pooled estimates. The confounders adjusted in the multivariate analysis for each study are listed in Supplementary Table 2. Next, subgroup analyses were made regarding the association between HLB (assessed with VSS 3–4 or FS ≥2) and poor functional outcome at 90 days based on the adjustments of important confounders, including age, baseline stroke severity (indicated with baseline NIHSS score), baseline collateral grade, stroke onset to recanalization time, and recanalization outcome after MT (Supplementary Table 5). Based on these results of subgroup analyses, we found that the association between HLB and poor functional outcome at 90 days remains stable.

The leukoaraiosis assessment strategy among the included studies was considered a key confounder for pooled estimates. Thus, we performed subgroup analyses for the association between HLB and poor functional outcome based on this key confounder: VSS 3–4 (OR 3.22, 95% CI 2.09–4.97; *p* < 0.001; 6 studies; *I*^2^ = 31.40%) and FS ≥2 (OR 2.11, 95% CI 1.44–3.10; *p* < 0.001; 4 studies; *I*^2^ = 0.00%) were found to be associated with an increased risk of poor functional outcome with low heterogeneity (Supplementary Figure 1 and Supplementary Table 5). Moreover, the variable recanalization rates among the included studies were also considered a key confounder. Next, subgroup analyses based on recanalization rate (categorized into >50% and ≤50%) indicated a stable association between HLB and poor functional outcome: >50% (OR 2.27, 95% CI 1.69–3.04; *p* < 0.001; 7 studies; *I*^2^ = 0.00%) and ≤50%) (OR 3.68, 95% CI 1.78–7.60; *p* < 0.001; 2 studies; *I*^2^ = 0.00%) (Supplementary Table 5). Meta-regression analyses were also conducted to explore the potential sources of heterogeneity. Adjustments for baseline collateral grade (*P*_interaction_ = 0.047) and the variable recanalization rates among the included studies (*P*_interaction_ = 0.053) accounted for the main heterogeneity among the included studies (Supplementary Table 5).

### Assessment of Publication Bias

We identified asymmetric funnel plots involved in the pooled estimates (associations between HLB and poor functional outcome at 90 days) (Supplementary Figure 2). Egger's tests also showed publication bias in this pooled estimate. Thus, the publication bias was adjusted through “trim and fill” analyses. This pooled estimate remained stable after the adjusted analysis (Supplementary Table 6).

## Discussion

Based on the results provided by 20 eligible studies, including ~5,189 patients with AIS undergoing MT, we performed a systematic review and meta-analysis to investigate the associations between SVD subtypes (including leukoaraiosis, CMBs, and lacunes) and outcomes of MT. The results showed that the presence of both HLB and CMBs at the time of AIS was associated with an increased risk of an unfavorable functional outcome at 90 days after MT, which indicated that patients with HLB and CMBs undergoing MT may have a higher risk of experiencing poor functional recovery than patients without HLB and CMBs. Moreover, we found that HLB was associated with a higher risk of in-hospital death, FR, and END. Our study demonstrated that CMBs were associated with a high risk of death at 90 days after MT. We also provided evidence of the safety of MT and evaluated the risks of post-MT ICH and sICH in patients with CMB burden. We found no significant association between CMBs and the risks of post-MT ICH and sICH.

With the development of MT for the treatment of AIS over the past several years, MT is now considered a standard therapy for AIS caused by LVO (5). The prevalence of SVDs, especially high SVD burden, is higher in patients with AIS caused by LVO, possibly because the main risk factor for developing SVD is age. Elderly patients have a higher risk of developing SVD; moreover, elderly patients have a higher risk of having AIS caused by LVO arising from large artery atherosclerosis or cardioembolism. Thus, SVD may be more likely to occur in addition to AIS caused by LVO as a concomitant disease, which may lead to poorer functional outcomes (36). The close relationship between LVO and SVD underscores the need to pay careful attention to the role of SVD in patients with AIS caused by LVO who are undergoing MT. Our findings in this meta-analysis may critically aid in predicting the efficiency and safety of MT, particularly in patients with SVD. This may help select patients for MT and facilitate perioperative communication with patients or their relatives.

The mechanisms underlying the clinical relevance between SVD and the outcomes of MT are poorly understood. SVD is associated with myelin damage, disconnection of white matter fibers, and neuronal degeneration that adversely affects neuroplasticity, further impairing brain repair function during neurorehabilitation (2, 37). Thus, patients with SVD (HLB and CMBs) undergoing MT may have a higher risk of experiencing poor functional outcome or FR, even though LVO has been recanalized with MT. Importantly, SVD mainly results from cerebral microcirculation disturbances (20), which are associated with vascular endothelium dysfunction and blood–brain barrier (BBB) dysfunction, further impairing cerebral blood flow in response to neuronal activity and decreasing neuronal energy supply (37). Thus, SVD-related poor microcirculation status may reduce brain tissue resistance to ischemia, further increasing the volume of the infarct core and resulting in poor functional outcomes (20). Thus, therapeutic strategies that improve cerebral microcirculation may alleviate the adverse influence of SVD on the outcomes of MT.

In patients undergoing IVT, both HLB and CMBs were found to be linked to an increased risk of ICH, possibly due to the close association of SVD with disturbances of microcirculation and the BBB and accompanied by enhanced fibrinolytic activity caused by IVT (4, 38). However, we found no clinical link between SVD (HLB and CMBs) and post-MT ICH or sICH. Unlike IVT, which affects fibrinolytic activity, MT confers its therapeutic effect via a mechanical mechanism (5). MT has little effect on fibrinolytic activity. Therefore, HLB and CMBs were not associated with a higher risk of post-MT ICH or sICH. In clinical practice, IVT is considered an adjunctive therapy of MT for patients with AIS caused by LVO within 4.5 h after symptom onset (39). Thus, we considered IVT to be a key confounder affecting the association between SVD and outcomes of MT. We should have been able to note the influence of SVD on the outcomes of MT combined with IVT. However, most of the included studies did not report this information, and only one eligible study with a small sample size reported no association between CMBs and outcomes in patients with AIS undergoing MT combined with IVT (22). More high-quality, prospective studies are needed to assess the effect of pre-existing SVDs on the outcomes of MT combined with IVT.

Several limitations should be considered when interpreting the results of this meta-analysis. First, the leukoaraiosis assessment strategy varied among several of the included studies. Although a higher VSS score, higher FS score, and greater volume of leukoaraiosis can all indicate HLB, each is a different type of assessment strategy, and the varying use of these assessments among included studies likely reduced the strength of the meta-analysis results. To decrease the effect of this confounding factor, we performed subgroup analyses. When the pooled analysis was layered by the three different assessment strategies, the associations of HLB with 90-day poor functional outcome and mortality after MT remained stable. Second, most of the included studies were small in size, which may have reduced the strength of our results. Third, most of the included studies focused on leukoaraiosis. Based on our comprehensive review of the literature, only two studies with a small sample size to date have investigated the association between lacunes and outcomes of MT (17, 18). Thus, the influence of lacunes on the outcomes of MT remains unclear. Therefore, well-conducted prospective studies with larger sample sizes are needed to assess the influence of lacunes on the outcomes of MT. Fourth, the quality of the included studies varied, and only seven studies were considered high-quality studies based on the Newcastle–Ottawa scale score (Supplementary Table 3). More high-quality studies are needed to improve the precision of pooled estimates. Fifth, we hypothesized a volume (assessed with volumetry neuroimaging software)–response association between leukoaraiosis and outcomes of MT. Unfortunately, most of the included studies assessed the association between leukoaraiosis and outcomes of MT *via* dichotomous comparisons. Only two studies investigated the volume–response relationship between leukoaraiosis and outcomes of MT (16, 21). Thus, there were insufficient data to conduct a volume–response meta-analysis. The study performed by Boulouis and colleagues reported a volume–response relationship between WMH and functional outcome of MT: each 1-cm^3^ increase in WMH volume, detected with volumetric MRICro software, was associated with a 1.5 times increased risk of unfavorable functional outcome of MT (16). However, a study with a smaller sample size performed by Atchaneeyasakul and colleagues reported no volume–response relationship between WMH and outcomes of MT (21). In light of these inconsistent results, it remains unclear whether there is a volume–response relationship between SVD and outcomes of MT. Sixth, the protocol of this systematic review has not been registered in a registry database. Registration of a protocol of a systematic review may help us improve its quality; moreover, registration may help investigators assess whether a systematic review has already been initiated. Based on our comprehensive review of the literature at the last search, we found no similar systematic reviews.

Taken together, in patients with AIS undergoing MT, HLB was associated with increased risks of a 90-day unfavorable functional outcome, in-hospital death, and FR after MT. CMBs were associated with increased risk of a 90-day unfavorable functional outcome after MT. Based on our findings, more attention should be paid to SVD in patients with AIS undergoing MT. In future trials investigating the efficacy and safety of MT in patients with AIS, the variable SVD phenotypes among patients should be taken into account, which may affect the outcomes of MT. In this meta-analysis, all included studies were conducted with observational designs; thus, future analyses of randomized controlled trial data are needed to explore whether the treatment efficacy of MT is weakened in patients with different SVD phenotypes.

## Data Availability Statement

The original contributions presented in the study are included in the article/Supplementary Material, further inquiries can be directed to the corresponding author/s.

## Author Contributions

TX and HL contributed to study design and drafting of the article. TX and YW performed literature search and selection. TX and JY performed data acquisition, analysis, and interpretation. YW and YC performed statistical analysis. All authors contributed to the article and approved the submitted version.

## Conflict of Interest

The authors declare that the research was conducted in the absence of any commercial or financial relationships that could be construed as a potential conflict of interest.

## Abbreviations

ACC: anterior cerebral circulation
AIS: acute ischemic stroke
AT: aspiration technique
BBB: blood–brain barrier
CMBs: cerebral microbleeds
END: early neurological deterioration
ENI: early neurological improvement
FLAIR: fluid-attenuated inversion recovery
FR: futile recanalization
FS: Fazekas scale
GRE: gradient-recall echo
HLB: high leukoaraiosis burden
ICH: intracranial hemorrhage
IVT: intravenous thrombolysis
LVO: large vessel occlusion
MARS: the Microbleed Anatomical Rating Scale
mRS: modified Rankin Scale score
MT: mechanical thrombectomy
mTICI: modified thrombolysis in cerebral infarction
NA: not available
NIHSS: National Institutes of Health Stroke Scale
PCC: posterior cerebral circulation
sICH: symptomatic intracranial hemorrhage
SR: stent retriever
SRE: successful recanalization
SVD: small vessel disease
SWI: susceptibility-weighted imaging
VSS: van Swieten scale
WMH: white matter hyperintensity.

## References

[1] Cannistraro RJ Badi M Eidelman BH Dickson DW Middlebrooks EH Meschia JF. CNS small vessel disease: a clinical review. Neurology. (2019) 92:1146–56. 10.1212/wnl.0000000000007654 31142635 PMC6598791

[2] Wardlaw JM Smith C Dichgans M. Small vessel disease: mechanisms and clinical implications. Lancet Neurol. (2019) 18:684–96. 10.1016/s1474-4422(19)30079-1 31097385

[3] Georgakis MK Duering M Wardlaw JM Dichgans M. WMH and long-term outcomes in ischemic stroke: a systematic review and meta-analysis. Neurology. (2019) 92:e1298–e308. 10.1212/wnl.0000000000007142 30770431

[4] Charidimou A Shoamanesh A. Clinical relevance of microbleeds in acute stroke thrombolysis: comprehensive meta-analysis. Neurology. (2016) 87:1534–41. 10.1212/wnl.0000000000003207 27629086

[5] Roman LS Menon BK Blasco J Hernandez-Perez M Davalos A Majoie C. Imaging features and safety and efficacy of endovascular stroke treatment: a meta-analysis of individual patient-level data. Lancet Neurol. (2018) 17:895–904. 10.1016/s1474-4422(18)30242-4 30264728

[6] Stroup DF Berlin JA Morton SC Olkin I Williamson GD Rennie D. Meta-analysis of observational studies in epidemiology: a proposal for reporting. Meta-analysis Of Observational Studies in Epidemiology (MOOSE) group. JAMA. (2000) 283:2008–12. 10.1001/jama.283.15.2008 10789670

[7] Stang A. Critical evaluation of the Newcastle-Ottawa scale for the assessment of the quality of nonrandomized studies in meta-analyses. Eur J Epidemiol. (2010) 25:603–5. 10.1007/s10654-010-9491-z 20652370

[8] Benson J Seyedsaadat SM Mark I Nasr DM Rabinstein AA Kallmes DF. Leukoaraiosis and acute ischemic stroke: 90-day clinical outcome following endovascular recanalization, with proposed “L-ASPECTS”. J Neurointerv Surg. (2020) 13:384–9. 10.1136/neurintsurg-2020-015957 32487764

[9] Mutzenbach JS Müller-Thies-Broussalis E Killer-Oberpfalzer M Griessenauer CJ Hecker C Moscote-Salazar LR. Severe Leukoaraiosis is associated with poor outcome after successful recanalization of m1 middle cerebral artery occlusion strokes. Cerebrovasc Dis. (2020) 49:1–9. 10.1159/000508209 32535590

[10] Mistry EA Mistry AM Mehta T Arora N Starosciak AK La Rosa F. White matter disease and outcomes of mechanical thrombectomy for acute ischemic stroke. AJNR Am J Neuroradiol. (2020) 41:639–44. 10.3174/ajnr.A6478 32165366 PMC7144640

[11] Mikati AG Mandelbaum M Sapnar S Puri AS Silver B Goddeau RP Jr.. Impact of leukoaraiosis severity on the association of time to successful reperfusion with 90-day functional outcome after large vessel occlusion stroke. Transl Stroke Res. (2020) 11:39–49. 10.1007/s12975-019-00703-0 30980282 PMC6925352

[12] Mechtouff L Nighoghossian N Amaz C Buisson M Berthezene Y Derex L. White matter burden does not influence the outcome of mechanical thrombectomy. J Neurol. (2020) 267:618–24. 10.1007/s00415-019-09624-2 31705292

[13] Guo Y Zhang S Li M Sun B Shang X Li S. Leukoaraiosis and earlier neurological outcome after mechanical thrombectomy in acute ischemic stroke. J Neuroradiol. (2020) 47:428–32. 10.1016/j.neurad.2019.10.005 32035971

[14] Liu Y Gong P Sun H Zhang S Zhou J Zhang Y. Leukoaraiosis is associated with poor outcomes after successful recanalization for large vessel occlusion stroke. Neurol Sci. (2019) 40:585–91. 10.1007/s10072-018-3698-2 30612278

[15] Guo Y Zi W Wan Y Zhang S Sun B Shang X. Leukoaraiosis severity and outcomes after mechanical thrombectomy with stent-retriever devices in acute ischemic stroke. J Neurointerv Surg. (2019) 11:137–40. 10.1136/neurintsurg-2018-014018 30045947

[16] Boulouis G Bricout N Benhassen W Ferrigno M Turc G Bretzner M. White matter hyperintensity burden in patients with ischemic stroke treated with thrombectomy. Neurology. (2019) 93:e1498–e506. 10.1212/wnl.0000000000008317 31519778 PMC6815208

[17] Arba F Testa GD Limbucci N Nappini S Renieri L Pracucci G. Small vessel disease and clinical outcomes after endovascular treatment in acute ischemic stroke. Neurol Sci. (2019) 40:1227–35. 10.1007/s10072-019-03824-4 30874998

[18] Sillanpaa N Pienimaki JP Protto S Seppanen J Numminen H Rusanen H. Chronic infarcts predict poor clinical outcome in mechanical thrombectomy of sexagenarian and older patients. J Stroke Cerebrovasc Dis. (2018) 27:1789–95. 10.1016/j.jstrokecerebrovasdis.2018.02.012 29525077

[19] Choi KH Kim JH Kang KW Kim JT Choi SM Lee SH. Impact of microbleeds on outcome following recanalization in patients with acute ischemic stroke. Stroke. (2019) 50:127–134. 10.1161/strokeaha.118.023084 30580721

[20] Gilberti N Gamba M Premi E Costa A Vergani V Delrio I. Leukoaraiosis is a predictor of futile recanalization in acute ischemic stroke. J Neurol. (2017) 264:448–52. 10.1007/s00415-016-8366-y 28004198

[21] Atchaneeyasakul K Leslie-Mazwi T Donahue K Giese AK Rost NS. White matter hyperintensity volume and outcome of mechanical thrombectomy with stentriever in acute ischemic stroke. Stroke. (2017) 48:2892–94. 10.1161/strokeaha.117.018653 28887393 PMC5659291

[22] Shi ZS Duckwiler GR Jahan R Tateshima S Gonzalez NR Szeder V. Mechanical thrombectomy for acute ischemic stroke with cerebral microbleeds. J Neurointerv Surg. (2016) 8:563–7. 10.1136/neurintsurg-2015-011765 25994939

[23] Giurgiutiu DV Yoo AJ Fitzpatrick K Chaudhry Z Leslie-Mazwi T Schwamm LH. Severity of leukoaraiosis, leptomeningeal collaterals, and clinical outcomes after intra-arterial therapy in patients with acute ischemic stroke. J Neurointerv Surg. (2015) 7:326–30. 10.1136/neurintsurg-2013-011083 24721756

[24] Zhang J Puri AS Khan MA Goddeau RP Jr. Henninger N. Leukoaraiosis predicts a poor 90-day outcome after endovascular stroke therapy. AJNR Am J Neuroradiol. (2014) 35:2070–5. 10.3174/ajnr.A4029 24994827 PMC7965186

[25] Gratz PP El-Koussy M Hsieh K von Arx S Mono ML Heldner MR. Preexisting cerebral microbleeds on susceptibility-weighted magnetic resonance imaging and post-thrombolysis bleeding risk in 392 patients. Stroke. (2014) 45:1684–8. 10.1161/strokeaha.114.004796 24743433

[26] Soize S Barbe C Kadziolka K Estrade L Serre I Pierot L. Predictive factors of outcome and hemorrhage after acute ischemic stroke treated by mechanical thrombectomy with a stent-retriever. Neuroradiology. (2013) 55:977–87. 10.1007/s00234-013-1191-4 23644538

[27] Shi ZS Loh Y Liebeskind DS Saver JL Gonzalez NR Tateshima S. Leukoaraiosis predicts parenchymal hematoma after mechanical thrombectomy in acute ischemic stroke. Stroke. (2012) 43:1806–11. 10.1161/strokeaha.111.649152 22581819 PMC3383878

[28] van Swieten JC Hijdra A Koudstaal PJ van Gijn J. Grading white matter lesions on CT and MRI: a simple scale. J Neurol Neurosurg Psychiatry. (1990) 53:1080–3. 10.1136/jnnp.53.12.1080 2292703 PMC488320

[29] Fazekas F Chawluk JB Alavi A Hurtig HI Zimmerman RA. MR signal abnormalities at 1.5 T in Alzheimer's dementia and normal aging. AJR Am J Roentgenol. (1987) 149:351–6. 10.2214/ajr.149.2.351 3496763

[30] Gregoire SM Chaudhary UJ Brown MM Yousry TA Kallis C Jager HR. The Microbleed Anatomical Rating Scale (MARS): reliability of a tool to map brain microbleeds. Neurology. (2009) 73:1759–66. 10.1212/WNL.0b013e3181c34a7d 19933977

[31] von Kummer R Broderick JP Campbell BC Demchuk A Goyal M Hill MD. The heidelberg bleeding classification: classification of bleeding events after ischemic stroke and reperfusion therapy. Stroke. (2015) 46:2981–6. 10.1161/strokeaha.115.010049 26330447

[32] Higashida RT Furlan AJ Roberts H Tomsick T Connors B Barr J. Trial design and reporting standards for intra-arterial cerebral thrombolysis for acute ischemic stroke. Stroke. (2003) 34:e109–37. 10.1161/01.str.0000082721.62796.09 12869717

[33] Higgins JP Thompson SG Deeks JJ Altman DG. Measuring inconsistency in meta-analyses. BMJ. (2003) 327:557–60. 10.1136/bmj.327.7414.557 12958120 PMC192859

[34] Egger M Davey Smith G Schneider M Minder C. Bias in meta-analysis detected by a simple, graphical test. BMJ. (1997) 315:629–34. 10.1136/bmj.315.7109.629 9310563 PMC2127453

[35] Duval S Tweedie R. Trim and fill: a simple funnel-plot-based method of testing and adjusting for publication bias in meta-analysis. Biometrics. (2000) 56:455–63. 10.1111/j.0006-341x.2000.00455.x 10877304

[36] Zhai FF Yang M Wei Y Wang M Gui Y Han F. Carotid atherosclerosis, dilation, and stiffness relate to cerebral small vessel disease. Neurology. (2020) 94:e1811–e19. 10.1212/wnl.0000000000009319 32241954

[37] O'Sullivan M. Imaging small vessel disease: lesion topography, networks, and cognitive deficits investigated with MRI. Stroke. (2010) 41:S154–8. 10.1161/strokeaha.110.595314 20876494

[38] Kongbunkiat K Wilson D Kasemsap N Tiamkao S Jichi F Palumbo V. Leukoaraiosis, intracerebral hemorrhage, and functional outcome after acute stroke thrombolysis. Neurology. (2017) 88:638–45. 10.1212/wnl.0000000000003605 28130468 PMC5317383

[39] Powers WJ Rabinstein AA Ackerson T Adeoye OM Bambakidis NC Becker K. Guidelines for the early management of patients with acute ischemic stroke:2019 update to the 2018 guidelines for the early management of acute ischemic stroke: a guideline for healthcare professionals from the American Heart Association/American Stroke Association. Stroke. (2019) 50:e344–e418. 10.1161/str.0000000000000211 31662037

