# Supplementary material for: Small Vessel Disease Burden and Outcomes of Mechanical Thrombectomy in Ischemic Stroke: A Systematic Review and Meta-Analysis
Source: Front Neurol. 2021 Apr 7;12:602037. doi: 10.3389/fneur.2021.602037 (PMC8058474; doi:10.3389/fneur.2021.602037)
Supplement: Supplementary file 1 [file Data_Sheet_1.PDF]

Supplementary materials

Companion to: Small vessel disease burden and outcomes of mechanical thrombectomy in ischemic stroke: a systematic review and meta-analysis

Table S1, page 2

Table S2, page 3-5

Table S3, page 6

Table S4, page 7

Table S5, page 8

Figure S1, page 9

Figure S2, page 10

Table S6, page 10

Table S1. Search strategy in the Medline database.

Steps	Queries	Number of studies
**#1**	Search (((((((((((((perivascular spaces) OR white matter hyperintensities) OR white matter lacunar) OR leukoaraiosis) OR small vessel disease) OR microbleeds) OR virchow-robin spaces) OR virchow robin spaces) OR lacunes) OR lacunar) OR white matter) OR perivascular) OR virchow-robin) OR microbleed) OR small vessel	156,985
**#2**	Search (((((Thrombectomy) OR Endovascular) OR reperfusion) OR Recanalization) OR Aspiration) OR retriever	288,047
**#1 and #2**	Search (((((((((((((((perivascular spaces) OR white matter hyperintensities) OR white matter lacunar) OR leukoaraiosis) OR small vessel disease) OR microbleeds) OR virchow-robin spaces) OR virchow robin spaces) OR lacunes) OR lacunar) OR white matter) OR perivascular) OR virchow-robin) OR microbleed) OR small vessel)) AND ((((((Thrombectomy) OR Endovascular) OR reperfusion) OR Recanalization) OR Aspiration) OR retriever)	5,031

The search strategy for the Embase and the Cochrane Library database was similar to that used for the Medline database

Table S2. The definitions of small vessel disease subtypes and the confounders adjusted for the multivariate analysis in each included study.

First Author, y of publication	The definitions of HLB, CMB burden, and lacune burden	Adjusted factors in each included study
Benson 2020(1)	Fazekas scale ≥ 2	NA
Mutzenbach 2020(2)	Top 25% based on ARWMC scale*	Baseline NIHSS score, sex, recanalization outcome after MT, pre-stroke mRS, and baseline collateral grade
Mistry 2020(3)	van Swieten 3-4	Age, hypertension, baseline glucose level, baseline SBP, baseline NIHSS score, onset-to-recanalization time, bridging intravenous thrombolysis, baseline ASPECTS, and recanalization outcome after MT
Mikati 2020(4)	van Swieten 3-4	NA
Mechtouff 2020(5)	Fazekas scale >2	NA
Guo 2020(6)	van Swieten 3-4	Age, baseline SBP, history of coronary heart diseases, baseline NIHSS score, baseline ASPECTS, and baseline collateral grade
Liu 2019(7)	Fazekas scale ≥ 2	Age, sex, baseline NIHSS score, onset-to-recanalization time, baseline serum creatinine, and baseline fasting glucose, smoking history, and baseline international normalized ratio
Guo 2019(8)	van Swieten 3-4	Age, baseline SBP, baseline NIHSS score, the etiology of stroke, baseline collateral grade, recanalization outcome after MT, and history of coronary heart diseases

Boulouis 2019(9)	High WMH volume	Age, sex, baseline NIHSS score, and onset-to-groin puncture time, and recanalization outcome after MT
Arba 2019(10)	van Swieten 3-4 and Lacune presence#	Age, sex, baseline NIHSS score, and onset-to-groin puncture time
Sillanpaa 2018(11)	Lacune presence#	NA
Choi 2018(12)	CMB presence detected with an MRI scan	Age, sex, hypertension, diabetes mellitus, dyslipidemia, atrial fibrillation, smoking history, stroke or transient ischemic attack history, pre-stroke modified Rankin Scale, baseline NIHSS score, baseline infarct volume, and recanalization outcome after MT
Gilberti 2017(13)	van Swieten 2-4	NA
Atchaneeyasakul 2017(14)	High WMH volume	baseline NIHSS score, onset-to-recanalization time, histroy of congestive heart failure, and recanalization outcome after MT
Shi 2016(15)	CMB presence detected with an MRI scan	Age, baseline NIHSS score, hypertension, diabetes mellitus, atrial fibrillation, baseline glucose level, baseline SBP, and onset-to-groin puncture time>5 h
Giurgiutiu 2015(16)	High WMH volume	Age, baseline NIHSS score, baseline glucose level, baseline diffusion weighted imaging outcome, recanalization outcome after MT, bridging intravenous thrombolysis, and hypertension
Zhang 2014(17)	van Swieten 3-4	Age, baseline NIHSS score, atrial fibrillation, baseline serum creatinine, dyslipidemia, baseline glucose level, recanalization outcome

		after MT, onset-to-recanalization time, baseline collateral grade, and parenchymal hemorrhage after MT
Gratz 2014(18)	CMB presence detected with an MRI scan	NA
Soize 2013(19)	Fazekas scale ≥ 2	NA
Shi 2012(20)	Fazekas scale ≥ 2	NA

Abbreviations: NA, not available; NIHSS, National Institutes of Health Stroke Scale; MT, mechanical thrombectomy; SBP, systolic blood pressure; ASPECTS, Alberta Stroke Program Early CT Score; WMH, white matter hyperintensity; CMBs, cerebral microbleeds.

*Leukoaraiosis severity assessed with Top 25% based on ARWMC scale (a semi-quantitative scale similar with VSS or FS)(2).

Lacune burden was defined as the presence of multiple lacunes (≥ 2) detected with a CT or MRI scan (10).

Table S3. Quality assessment of the included studies*

Reference#	Is the exposed cohort representative?	Selection of the non-exposed cohort	Ascertainment of exposure	Demonstration that outcome of interest was not present at start of study	Comparability of important factors†	Assessment of outcome	Follow up period	Adequacy of follow up of cohorts	Total quality scores
Benson 2020(1)	☆	☆	☆	☆	☆	☆	☆	☆	8
Mutzenbach 2020(2)	☆	☆	☆	☆	☆	☆	☆	☆	8
Mistry 2020(3)	☆	☆	☆	☆	☆	☆	☆	☆	8
Mikati 2020(4)	☆	☆	☆	☆	—	☆	☆	—	6
Mechtouff 2020(5)	☆	☆	☆	☆	☆	☆	—	—	6
Guo 2020(6)	☆	—	☆	☆	☆ ☆	☆	—	—	6
Liu 2019(7)	☆	☆	☆	☆	☆ ☆	☆	—	—	7
Guo 2019(8)	☆	☆	☆	☆	☆ ☆	☆	—	—	7
Boulouis 2019(9)	☆	—	☆	☆	☆ ☆	☆	☆	☆	8
Arba 2019(10)	☆	☆	☆	☆	—	☆	—	—	5
Sillanpaa 2018(11)	☆	☆	☆	☆	—	☆	—	—	5
Choi 2018(12)	☆	☆	☆	☆	☆ ☆	☆	☆	☆	9
Gilberti 2017(13)	☆	☆	☆	☆	—	☆	—	—	5
Atchaneeyasakul(14)	☆	☆	☆	☆	—	☆	—	—	5
Shi 2016(15)	☆	☆	☆	☆	☆ ☆	☆	☆	☆	9
Giurgiutiu 2015(16)	☆	—	☆	☆	☆ ☆	☆	—	—	6
Zhang 2014(17)	☆	☆	☆	☆	—	☆	—	—	5
Gratz 2014(18)	☆	☆	☆	☆	—	☆	—	—	5
Soize 2013(19)	☆	☆	☆	☆	—	☆	—	—	5
Shi 2012(20)	☆	☆	☆	☆	☆	☆	☆	☆	8

*Newcastle-Ottawa Scale was used to assess the study quality in this meta-analysis(21). The full score was 9 stars, and the high-quality study was defined as a study with 8 awarded stars. †A maximum of two stars could be awarded for this item. One star with adjustment for age, two stars if there were additional population demographics or comorbidities.

Table S4. Meta-analysis of the association between SVD subtypes and outcomes of MT.

**Outcomes**	**Number of studies**	**Sample size**	**OR**	**95% CI**	**p-values**	I^2
**HLB (VSS 3-4 or FS ≥ 2) and outcomes of MT**						
90-day poor functional outcome*(3-5,7,8,10,17,19,1,2)	10	2,004	2.70	2.01–3.63	< 0.001	18.90%
90-day mortality(3,7,8,19)	4	818	1.59	0.99-2.55	0.056	0.00%
In-hospital death*(2,20)	2	314	4.06	1.48–11.13	0.006	54.00%
Post-MT ICH(5,20,1,2)	4	697	1.07	0.53–2.14	0.858	67.30%
Post-MT sICH(3,7,8,19,2)	5	1,005	1.81	1.00–3.29	0.051	0.00%
Successful recanalization (3,5)	2	598	1.16	0.73-1.86	0.528	0.00%
Futile recanalization*(8,13,1)	3	493	5.00	2.86–8.73	< 0.001	0.00%
Early neurological improvement(3,6)	2	662	0.58	0.19-1.72	0.324	77.80%
Early neurological deterioration*(6)	1	273	2.65	1.09-6.45		
**HLB (volumetric software) and outcomes of MT**						
90-day poor functional outcome#(9,14,16)	3	625	1.04	1.02-1.07	0.001	0.00%
90-day mortality(9,14)	2	552	1.13	0.79-1.62	0.515	45.4%
Post-MT ICH(14)	1	56	1.06	0.60-1.87		
Post-MT sICH(9)	1	496	0.99	0.93-1.04		
Successful recanalization(14)	1	56	1.30	0.70-2.38		
**CMBs and outcomes of MT**						
90-day poor functional outcome&(12,18)	2	1,924	1.84	1.17-2.90	0.008	0.00%
90-day mortality(18)	1	392	2.28	1.04-4.99		
In-hospital death(15)	1	206	0.57	0.17-1.84		
Post-MT ICH(15,18)	2	598	0.60	0.27-1.32	0.204	0.00%
Post-MT sICH(12,18)	2	1,924	1.42	0.19-10.55	0.733	69.20%
**Lacunes and outcome of MT**						
90-day poor functional outcome(10,11)	2	243	2.23	0.95-5.22	0.066	0.00%

Abbreviations: OR, odds ratio; CI, confidence interval; MT, mechanical thrombectomy; HLB, high leukoaraiosis burden; ICH, intracranial hemorrhage; sICH, symptomatic intracranial hemorrhage; CMBs, cerebral microbleeds.

*p < 0.007 was considered statistically significant based on Bonferroni corrections.

#p < 0.010 was considered statistically significant based on Bonferroni corrections.

&p < 0.010 was considered statistically significant based on Bonferroni corrections.

Table S5. Subgroup analyses regarding the association between HLB (assessed with VSS 3-4 or FS ≥ 2) and poor functional outcome at 90 days.

Variables	Number of studies	Sample size	OR	95% CI	p-values	I^2	PI
Assessment scales							0.237
VSS 3-4	6	1381	3.22	2.09-4.97	p < 0.001	31.40%	
FS ≥ 2	4	623	2.11	1.44-3.10	p < 0.001	0.00%	
Adjusted for confounders							0.328
Yes	6	1334	3.14	2.05-4.82	p < 0.001	28.20%	
No	4	670	2.18	1.49-3.18	p < 0.001	0.00%	
Adjusted for age							0.418
Yes	5	1041	3.18	1.90-5.33	p < 0.001	46.20%	
No	5	963	2.28	1.60-3.25	p < 0.001	0.00%	
Adjusted for baseline NIHSS score							0.328
Yes	6	1334	3.14	2.05-4.82	p < 0.001	28.20%	
No	4	670	2.18	1.49-3.18	p < 0.001	0.00%	
Adjusted for baseline collateral grade							0.047
Yes	2	380	5.25	2.77-9.93	p < 0.001	0.00%	
No	8	1624	2.27	1.70-3.02	p < 0.001	0.00%	
Adjusted for stroke onset to recanalization time							0.184
Yes	3	615	3.77	2.24-6.34	p < 0.001	0.00%	
No	7	1389	2.34	1.70-3.23	p < 0.001	9.50%	
Adjusted for recanalization outcome after MT							0.240
Yes	3	811	3.59	2.17-5.95	p < 0.001	0.10%	
No	7	1193	2.38	1.70-3.33	p < 0.001	14.10%	
Recanalization rate							0.053
>50%	7	437	2.27	1.69-3.04	p < 0.001	0.00%	
≤50%	2	1438	3.68	1.78-7.60	p < 0.001	0.00%	

Abbreviations: OR, odds ratio; CI, confidence interval; MT, mechanical thrombectomy; PI, P interaction.; HLB, high leukoaraiosis burden; NIHSS, National Institutes of Health Stroke Scale.

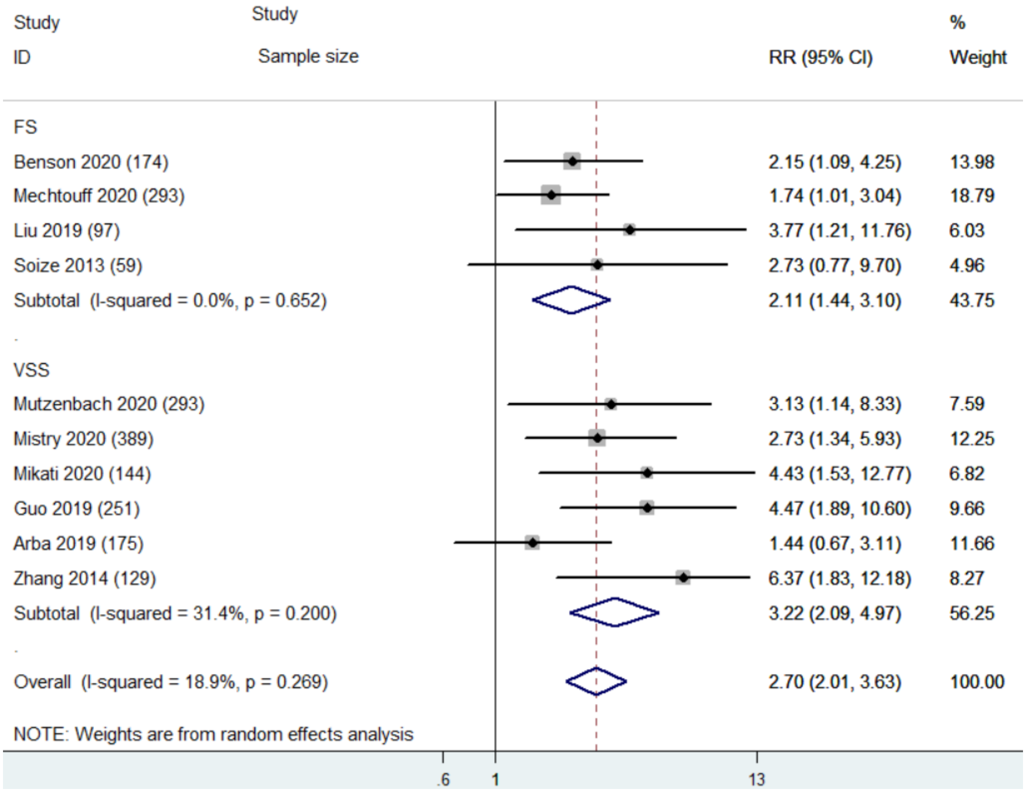

Figure S1. Subgroup analyses for the association between high leukoaraiosis burden and poor functional outcome at 90 day after mechanical thrombectomy based on variable leukoaraiosis assessment strategies: van Swieten scale 3-4 and Fazekas scale ≥ 2.

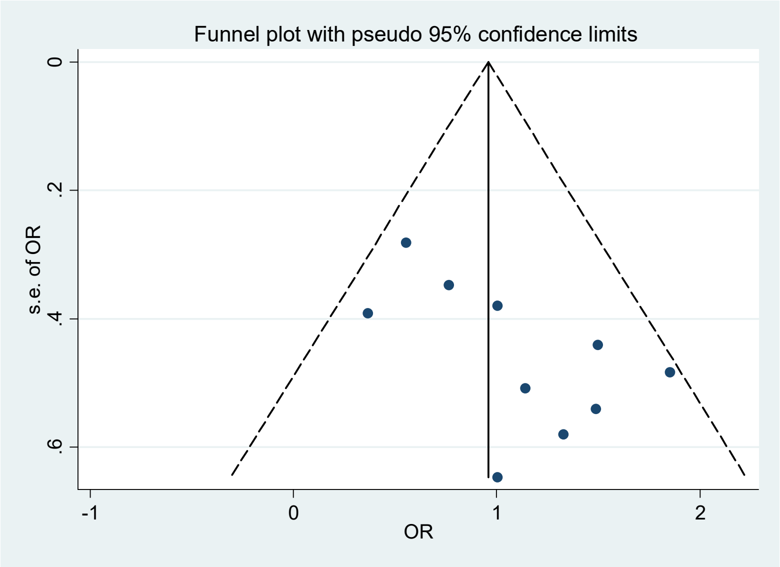

Figure S2. Funnel plot of pooled estimate for the association between high leukoaraiosis burden and poor functional outcome at 90 day after mechanical thrombectomy.

Table S6. Egger's tests and "trim and fill" analyses for publication bias.

**Variables**	**Egger's tests**		**Adjusted pooled ORs and 95% CIs from "trim and fill" analyses**
	**p-values**	**95% CIs**	
Association between HLB and poor functional outcome at 90 days(3-5,7,8,10,17,19,1,2)	0.037	0.23-5.50	2.11(1.51-2.94)

Abbreviations: CI, confidence interval; OR, odds ratio; HLB, high leukoaraiosis burden.

References

1. Benson J, Seyedsaadat SM, Mark I, Nasr DM, Rabinstein AA, Kallmes DF et al. Leukoaraiosis and acute ischemic stroke: 90-day clinical outcome following endovascular recanalization, with proposed "L-ASPECTS". J Neurointerv Surg. (2020). doi:10.1136/neurintsurg-2020-015957

2. Mutzenbach JS, Müller-Thies-Broussalis E, Killer-Oberpfalzer M, Griessenauer CJ, Hecker C, Moscote-Salazar LR et al. Severe Leukoaraiosis Is Associated with Poor Outcome after Successful Recanalization of M1 Middle Cerebral Artery Occlusion Strokes. Cerebrovasc Dis. (2020) 1-9. doi:10.1159/000508209

3. Mistry EA, Mistry AM, Mehta T, Arora N, Starosciak AK, La Rosa F et al. White Matter Disease and Outcomes of Mechanical Thrombectomy for Acute Ischemic Stroke. AJNR Am J Neuroradiol. (2020). doi:10.3174/ajnr.A6478

4. Mikati AG, Mandelbaum M, Sapnar S, Puri AS, Silver B, Goddeau RP, Jr. et al. Impact of

Leukoaraiosis Severity on the Association of Time to Successful Reperfusion with 90-Day Functional Outcome After Large Vessel Occlusion Stroke. Transl Stroke Res. (2020) 11: 39-49. doi:10.1007/s12975-019-00703-0
5. Mechtouff L, Nighoghossian N, Amaz C, Buisson M, Berthezene Y, Derex L et al. White matter burden does not influence the outcome of mechanical thrombectomy. J Neurol. (2020) 267: 618-24. doi:10.1007/s00415-019-09624-2
6. Guo Y, Zhang S, Li M, Sun B, Shang X, Li S et al. Leukoaraiosis and earlier neurological outcome after mechanical thrombectomy in acute ischemic stroke. J Neuroradiol. (2020). doi:10.1016/j.neurad.2019.10.005
7. Liu Y, Gong P, Sun H, Zhang S, Zhou J, Zhang Y. Leukoaraiosis is associated with poor outcomes after successful recanalization for large vessel occlusion stroke. Neurol Sci. (2019) 40: 585-91. doi:10.1007/s10072-018-3698-2
8. Guo Y, Zi W, Wan Y, Zhang S, Sun B, Shang X et al. Leukoaraiosis severity and outcomes after mechanical thrombectomy with stent-retriever devices in acute ischemic stroke. J Neurointerv Surg. (2019) 11: 137-40. doi:10.1136/neurintsurg-2018-014018
9. Boulouis G, Bricout N, Benhassen W, Ferrigno M, Turc G, Bretzner M et al. White matter hyperintensity burden in patients with ischemic stroke treated with thrombectomy. Neurology. (2019) 93: e1498-e506. doi:10.1212/wnl.0000000000008317
10. Arba F, Testa GD, Limbucci N, Nappini S, Renieri L, Pracucci G et al. Small vessel disease and clinical outcomes after endovascular treatment in acute ischemic stroke. Neurol Sci. (2019) 40: 1227-35. doi:10.1007/s10072-019-03824-4
11. Sillanpaa N, Pienimaki JP, Protto S, Seppanen J, Numminen H, Rusanen H. Chronic Infarcts Predict Poor Clinical Outcome in Mechanical Thrombectomy of Sexagenarian and Older Patients. J Stroke Cerebrovasc Dis. (2018) 27: 1789-95. doi:10.1016/j.jstrokecerebrovasdis.2018.02.012
12. Choi KH, Kim JH, Kang KW, Kim JT, Choi SM, Lee SH et al. Impact of Microbleeds on Outcome Following Recanalization in Patients With Acute Ischemic Stroke. Stroke. (2018) STROKEAHA118023084. doi:10.1161/strokeaha.118.023084
13. Gilberti N, Gamba M, Premi E, Costa A, Vergani V, Delrio I et al. Leukoaraiosis is a predictor of futile recanalization in acute ischemic stroke. J Neurol. (2017) 264: 448-52. doi:10.1007/s00415-016-8366-y
14. Atchaneeyasakul K, Leslie-Mazwi T, Donahue K, Giese AK, Rost NS. White Matter Hyperintensity Volume and Outcome of Mechanical Thrombectomy With Stentriever in Acute Ischemic Stroke. Stroke. (2017) 48: 2892-94. doi:10.1161/strokeaha.117.018653
15. Shi ZS, Duckwiler GR, Jahan R, Tateshima S, Gonzalez NR, Szeder V et al. Mechanical thrombectomy for acute ischemic stroke with cerebral microbleeds. J Neurointerv Surg. (2016) 8: 563-7. doi:10.1136/neurintsurg-2015-011765
16. Giurgiutiu DV, Yoo AJ, Fitzpatrick K, Chaudhry Z, Leslie-Mazwi T, Schwamm LH et al. Severity of leukoaraiosis, leptomeningeal collaterals, and clinical outcomes after intra-arterial therapy in patients with acute ischemic stroke. J Neurointerv Surg. (2015) 7: 326-30. doi:10.1136/neurintsurg-2013-011083
17. Zhang J, Puri AS, Khan MA, Goddeau RP, Jr., Henninger N. Leukoaraiosis predicts a poor 90-day outcome after endovascular stroke therapy. AJNR Am J Neuroradiol. (2014) 35: 2070-5. doi:10.3174/ajnr.A4029
18. Gratz PP, El-Koussy M, Hsieh K, von Arx S, Mono ML, Heldner MR et al. Preexisting cerebral

microbleeds on susceptibility-weighted magnetic resonance imaging and post-thrombolysis bleeding risk in 392 patients. Stroke. (2014) 45: 1684-8. doi:10.1161/strokeaha.114.004796
19. Soize S, Barbe C, Kadziolka K, Estrade L, Serre I, Pierot L. Predictive factors of outcome and hemorrhage after acute ischemic stroke treated by mechanical thrombectomy with a stent-retriever. Neuroradiology. (2013) 55: 977-87. doi:10.1007/s00234-013-1191-4
20. Shi ZS, Loh Y, Liebeskind DS, Saver JL, Gonzalez NR, Tateshima S et al. Leukoaraiosis predicts parenchymal hematoma after mechanical thrombectomy in acute ischemic stroke. Stroke. (2012) 43: 1806-11. doi:10.1161/strokeaha.111.649152
21. Stang A. Critical evaluation of the Newcastle-Ottawa scale for the assessment of the quality of nonrandomized studies in meta-analyses. Eur J Epidemiol. (2010) 25: 603-5. doi:10.1007/s10654-010-9491-z
